# Fecal microbiota characterization of an Italian local horse breed

**DOI:** 10.3389/fvets.2024.1236476

**Published:** 2024-02-15

**Authors:** Alicia Maria Carrillo Heredero, Alberto Sabbioni, Vittoria Asti, Michela Ablondi, Andrea Summer, Simone Bertini

**Affiliations:** Department of Veterinary Sciences, University of Parma, Parma, Italy

**Keywords:** horse, local breed, Bardigiano, microbiota, biodiversity

## Abstract

The Bardigiano horse is a traditional native Italian breed with a rich history and peculiar characteristics. Local breeds are proven to have unique genetic traits developed over generations to adapt to defined geographical regions and/or conditions. The specific microbial communities that coexist within these animals are unraveled by studying their microbiota, which permits a further step in the characterization of local heritage. This work aimed to characterize Bardigiano horse fecal microbiota composition. The data obtained were then compared with published data of a mix of athlete breeds to evaluate potential differences among local and specialized breeds. The study involved 11 Bardigiano mares between 3 and 4 years of age, from which stool was sampled for the study. Samples were processed for 16S rRNA sequencing. Data obtained were analyzed and plotted using R, RStudio, and FastTree software. The samples analyzed were similar to what literature has reported on horses of other breeds and attitudes at higher taxonomic levels (from phylum to genera). While at lower taxonomic levels, the difference was more marked highlighting specific families found in the Bardigiano breed only. Weight, province of origin, and breeding sites significantly affected microbiota composition (*p*-value ≤0.02, *p*-value ≤0.04, and *p*-value ≤0.05, respectively). The comparison with athlete breed showed a significant difference confirming that animal and environmental factors are crucial in determining fecal microbiota composition (*p*-value <0.001). Understanding the microbiota composition in local breeds like the Bardigiano horse is crucial for preserving biodiversity, managing animal health, and promoting sustainable farming practices.

## 1 Introduction

**Figure 1:**
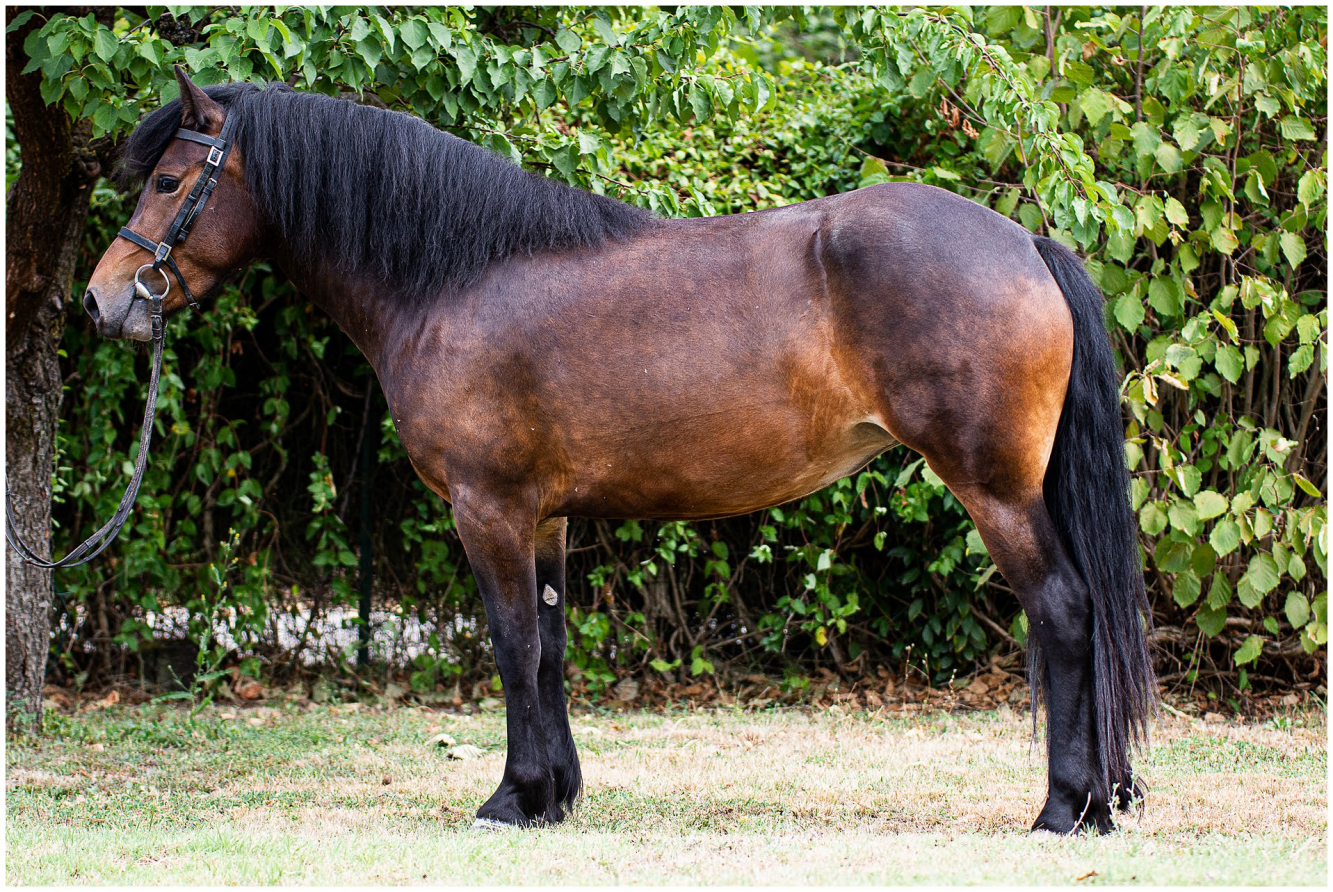
Example of Bardigiano horse. Picture by Francesca Rossi.

The Bardigiano horse, as represented in Figure 1, is a traditional native Italian breed with a rich history and peculiar characteristics. Its name is related to Bardi, a small town on the hill in the province of Parma, Italy. The Bardigiano breed played a crucial role in human society during the 19th century, serving as a means of transportation in agriculture as well as for meat production (1). Unfortunately, after the Second World War, the breed faced a major threat to its survival since only five stallions and 150 mares survived. To overcome this severe bottleneck, the Bardigiano studbook was founded in 1977 to preserve the breed’s unique features while improving its use for riding and draft purposes. Furthermore, recent strategies have been implemented in the breed both using pedigree and genotype data to reduce the loss of genetic diversity (2, 3). Nowadays, the Bardigiano breed counts roughly 3,000 live horses, mainly used for riding and light draft purposes. This breed is an example of a successful project to preserve local heritage through adaptation to the current market demand. This is possible through the implementation of breeding values as well as optimal contribution selection tools (2, 4).

To meet the market demand, the selection of this breed is leading it towards a meso-endomorph morphological type, characterized by moderate dimensions and a calm temperament. The distinctive traits of the breed can be summarized as follow: (a) the height ranges between 140 and 149 cm for males and 135 and 147 cm for females; (b) chest circumference higher than 165 cm; (c) the coat color is bay, with a preference for dark bay; and (d) limited white markings on the legs and face are allowed, although not preferred. Typical conformation includes a small head with a straight or concave profile, low withers, a slightly straight back, deep girth, and an overall muscular appearance. Bardigiano horses also show unique traits ranging from excellent resilience to harsh climates, roughage diet, pasture conditions, gentle temperament, and willingness to work (5).

Therefore, Bardigiano horses represent a unique breed with a deep history, cultural significance, and a current position in the market thanks to their peculiar characteristics. The genetic identity of the Bardigiano breed was likewise corroborated by a recent study where the Italian equine gene pool was analyzed by genotype data (6). Several research activities have been performed to fully characterize this breed from a genetic point of view; however, nothing is yet known about the microbiota of Bardigiano horses. By studying those horses’ microbiota, we might better understand their unique adaptations and potential environmental influences that have shaped the breed over time.

The microbiota is the microbial community that inhabits a certain environment or organ. Fecal microbiota composition has been widely described in human and major animal species (7–10). The characterization of the microbiota in local breeds is important since they are proven to have unique genetic traits developed over generations to adapt to specific geographical regions and/or conditions. By characterizing the microbiota, we better understand the specific microbial communities that coexist within these animals (11, 12). Therefore, studying the microbiota of native breeds might lead to a further step to unravel peculiar within-breed characteristics. Several works have used the characterization of microbiota as a tool to evaluate animals’ good health and resilience because of its tight links to the immune system (13, 14). It is proven that the more diverse the microbiome the more the probability of the individual being in good health. Moreover, knowing the breed microbiota composition allows for identifying any changes (dysbiosis) that could be caused by or lead to pathological states (15). Since the intestinal microbial community is involved in nutrient absorption, digestion, and building a strong immune response, its description has also a functional role. Understanding the microbiota can help target measures to promote animal wellness and prevent diseases. Studying microbiological diversity might help to enhance the repopulation of endangered breeds: since the microbiota is transmitted from the mother to the offspring, it allows to detect the healthiest individuals and potentially more resistant to adverse conditions (16). Therefore, that knowledge can help to preserve the biodiversity of local breeds and to ensure their long-term survival. The characterization of the microbiota in local breeds can also provide valuable insights into the genetic and phenotypic characteristics associated with specific microbial communities (17). By studying the interactions between host genetics, microbiota, and environmental factors, researchers can identify potential genetic markers or traits that are correlated with desirable microbial profiles. This information can be useful in selective breeding programs to improve animal health, productivity, and resilience (18). Characterization of local breeds and species has been encouraged in several international projects. The United Nations, as part of its Sustainable Development Goals outlined in the 2030 Agenda, has set ambitious targets aimed at preserving Life on Earth. These objectives encompass halting biodiversity loss, fostering genetic diversity, and championing the appreciation and valorization of local breeds (19).

Thus, the identification of the microbiota composition in local breeds is essential for preserving biodiversity, managing animal health and disease, promoting sustainable farming practices, and unlocking the potential for genetic and phenotypic improvements. Finally, it helps to understand the intricate relationship between animals and their microbial partners, leading to better strategies for animal welfare, conservation, and agricultural sustainability.

This work aimed to study the fecal microbial community in the Bardigiano horse breed, an Italian local breed originating in the hills of the province of Parma, to add a further step in its characterization. The study evaluated various aspects that potentially influence microbiota composition, considering both intrasubject characteristics as well as environmental factors acting in the development age or adulthood. In addition, the data obtained from Bardigiano horses were compared to the athletic breeds’ from Plancade et al. (20) to further disentangle potential differences between local breeds and cosmopolitan ones.

## 2 Materials and methods

### 2.1 Inclusion criteria

The 11 female horses, subscribed to the Bardigiano studbook, were selected by ANAREAI (National Breeders Association for Italian Equine and Asinine breeds), based on their age, breed standards, and biometrical measurements. All breeders were aware of this experiment as they agreed through ANAREAI to subject their animals to experiments for the characterization and valorization of the breed itself. A maximum of two animals for each breeder were allowed for a total of 10 breeders (6 at Site 1 and 4 at Site 2). Before starting the protocol, all horses underwent examinations by judges and veterinarians to ensure they did not have any pathologies or lameness. The areas where the 11 horses were born were classified into plains and hills, based on altitude following the guidelines from the Italian National Statistical Institute (ISTAT, 2018), where hills are defined as lands from 300 to 700 m and lowlands lower than 300 m from sea level. The horses were reared in two different riding centers (Site 1 and Site 2); feeding, training, and environmental conditions were identical within each riding center; however, there were differences comparing the conditions between the two riding centers. Site 1 is situated in a flatland in the province of Parma (Italy), at coordinates 44.93452935855489° N, 10.359899154478137° E, and hosts a total population of 50 horses with several other breeds. At this site, the horses have been subjected to a daily training session lasting 20 min, with 1 day of rest per week. Site 2 is situated in a hilly area in the province of Parma (Italy) at coordinates 44.5042826805862° N, 9.658427278379634° E; these stable counts more than 40 horses, and most of them belong to the Bardigiano breed. The tested horses at this site are housed all in the same stable, while the remaining ones have access to a large pasture. The training routine for the tested horses at Site 2 was a one-hour session every 2 days. A 70 days training protocol started, during which the eleven horses were trained by one single trainer in a conditioning stable. The diet of the horses was standardized among horses belonging to the study consisting of 12 kg of hay and 1.3 kg of concentrate for Site 1 and 15 kg of hay and 500 g of concentrate in Site 2. The quantity of the hay is similar between Site 1 and Site 2, although there are differences in their quality and origin due to different altitudes and therefore availability at the two sites. At Site 1, the hay originates from flatland locations, while, at Site 2, the hay is sourced from the hills surrounding the riding center, resulting in less variability in the diet of the horses. Both sites use concentrate feedstuff, although the specific type of feed differs between them. At Site 1, a supplementary feed indicated for the rehydration of horses during the summer months is used. Also, the bedding differs, in Site 1 wood flakes were used while in Site 2 straw.

### 2.2 Weight of horse

The weight of the horses was estimated according to Marcenac et al. (21), where weight expressed in kilograms = (chest circumference expressed in meters)^3^× 80. Three weight classes were established based on percentile divisions of the estimated weight within the samples. These classes were then assessed and analyzed in the study, as indicated in Supplementary Table S1.

### 2.3 Fecal sampling and 16S rRNA sequencing

The stool samples were taken in July 2022 after 70 days of conditioning protocol which can be considered as an “acclimation period” where the environmental conditions where set in the two sites as previously described in the “inclusion criteria paragraph.” Approximately 30 g of feces were collected and transferred in a sterile plastic flask. The samples were then transported in a cooler to the lab, where three aliquots of each sample were made in sterile cryotubes and stored at – 80°C until extraction.

The DNA of the samples was extracted as indicated by QIAamp UCP Pathogen Mini Kit purchased from QIAGEN (Hilden, Germany), including a blank control. The quantification of the DNA extracted was performed through qPCR of the 16S rRNA sequences (regions V3 and V4) which included a negative control consisting of a MOC Community and filtered sterile water after UV treatment. The library was set up using a Quick-16S NGS Library Prep Kit (Zymo 145 Research, Irvine, CA, United States) and analyzed using a MiSeq Reagent Kit v3 (600 cycles, 2 × 300 bp paired-end reads).

### 2.4 Data and statistical analysis

Using DADA2 pipeline v1.16 (22) in R v4.2.3 we processed reads obtained from sequencing. R packages used at this phase were dada2 and DECIPHER. Samples obtained were split into individual fastq files containing both forward and reverse reads. Firstly, using filterAndTrim function reads were filtered based on standard filtering parameters suggested by Edgar et al. (23), then the learnErrors function was used on filtered reads and errors were plotted through plotErrors function to visualize estimated error rates. The sequence data underwent filtering and trimming according to the inference algorithm from Callahan et al. (24), afterwards by means of mergePairs function forward and reverse reads were merged. We built an amplicon sequence variant (ASV) table using makeSequenceTable. Chimeras were then removed using the removeBimeraDenovo function. The sequences were classified according to the SILVA database version 138.1 (Small Subunit rRNA database) with the assignTaxonomy function (20). The quality control (QC) of the data was filtered based on ASV abundance (0.01%) per sample. Taxonomic features, beta diversity, and PairwiseAdonis test were performed using the following R packages: phyloseq, readxl, tibble, ape, MicrobiotaProcess, ggplot2, ggtree, plyr, vegan. Principal Coordinates Analysis (PCoA) allows for plotting ecological dissimilarity distances among microbial communities. Beta diversity was plotted using PCoA based on the Bray-Curtis distance index (Hellinger method) from the OTUs and taxa relative abundance table. A phylogenetic tree was created based on taxa identified using the open-source software FastTree and the following r Packages: DECIPHER, Biostrings, ape, adegenet, ggtree, ggtreeExtra, phyloseq, dplyr. The microbiota difference between horses kept in Site 1 and Site 2 was evaluated based on Welch’s *t*-test when two groups were present as in the case of place of origin, their altitude (flatland and hills), and when comparing the two sampling locations. Whereas, in the case of multiple levels within a factor (as in the case of estimated weight) a ANOVA was performed.

### 2.5 Comparison between Bardigiano and athletic breed

To further describe and distinguish the breed’s unique characteristics, the data found in this study was compared to published microbiota data from a pool of athlete breeds subjects. A random sample of 21 horses was downloaded from the open-source project BioProject PRJNA438436 by Plancade et al. (20). The horses, although from different breeds, ranging from Anglo-Arabian to Arabian, all performed endurance racing over 90 km. Therefore, they can be considered as a suited comparison to local breeds as they can be categorized as athlete horses. The data obtained in fastq format were processed using DADA2 pipeline v1.16 in R v4.2.3 and as described above. The relative abundance was compared between the two types of horses (Bardigiano and athletic one) and the ANOVA test was performed to detect potential breed differences in terms of taxa abundance and composition. The principal coordinate analysis was conducted based on the Bray-Curtis distance index.

## 3 Results and discussion

### 3.1 Taxonomic description

A total of 1,633,013 reads were obtained from the sequencing. After filtering we had 1,305,837 reads. After the assignments of the taxonomy in R, 14 phyla, 18 classes, 32 orders, 59 families, 118 genes and 132 species were found.

**Figure 2:**
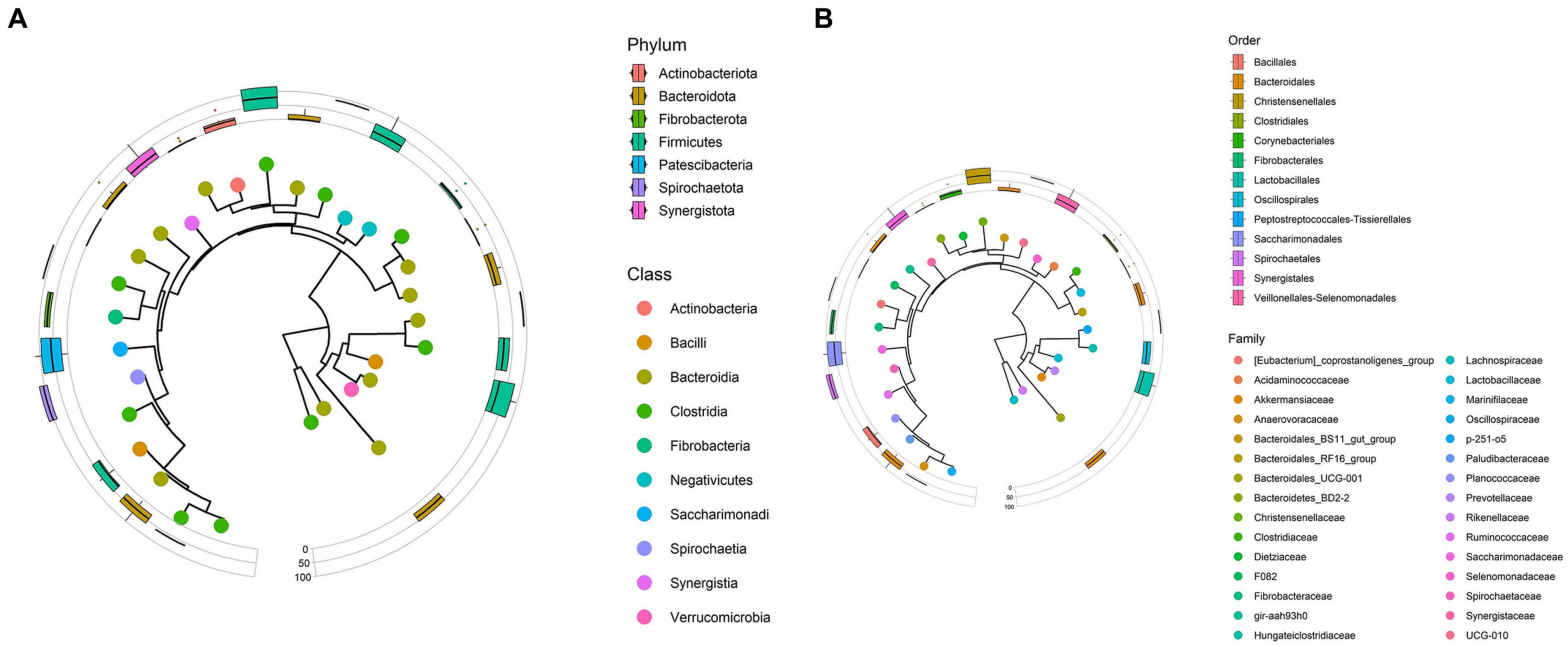
**(A)** Phylogenetic tree representing phyla and classes data observed from Bardigiano horses’ fecal samples. **(B)** Phylogenetic tree representing orders and families data observed from Bardigiano horses’ fecal samples.

The data showed that at the phylum level, the sample group was made on average mainly of Firmicutes (over 50%) and secondly of Bacteroidota (33.52%) and Spirochaeotota (6.27%), confirming published findings on horses’ microbiota (16, 25). Other phyla encountered were Fibrobacterota (3.36%) as shown in Figure 2A. These findings are in line with what has been reported by several authors in terms of taxa encountered in horse fecal microbiota studies (16, 18, 20, 25–27).

Taxa with the highest mean abundance regarding the class data were Clostridia (39.30%) and Bacteroida (33.52%), followed in smaller percentages by Bacilli (6.52%), and Spirochaetia (6.27%) as previously described by Mach et al. (16).

At the order level, as shown in Figure 2B, Bacterioidales covered 33.48% of relative abundance on average, followed by Oscillospirales (21.19%) and Lachnospirales (13.71%). A minor role is played by other orders, whose relative abundances, all under 6.5%, are reported in Supplementary Table S4.

Lachnospiraceae (13.63%), Oscillospiraceae (10.68%), Rikenellaceae (8.39%), Prevotellaceae (8.22%), F082 unclassified (6.47%), and Spirochaetaceae (6.27%) families, in respective decreasing order, composed, on average over 50% of the microbiota. Other families identified in the sample with an average relative abundance lower than 5% are reported in Supplementary Table S5.

Only Rikenellaceae RC9 gut group, F082 unclassified genus, and Treponema had abundance over 5% (respectively 7.52, 6.47, 6.25%). These three genera made up the 52.33% of relative abundance together with Streptococcus (4.63%), Lachnospiraceae AC2044 group (4.57%), NK4A214 group (4.38%), p-251-o5 unclassified genus (4.33%), Phascolarctobacterium (3.83%), UCG-010 unclassified genus (3.49%), Lachnospiraceae unclassified genus (3.49%), Fibrobacter (3.36%).

The list of all taxa is provided with relative abundance in Supplementary Table S7.

### 3.2 Factors of influence

**Figure 3:**
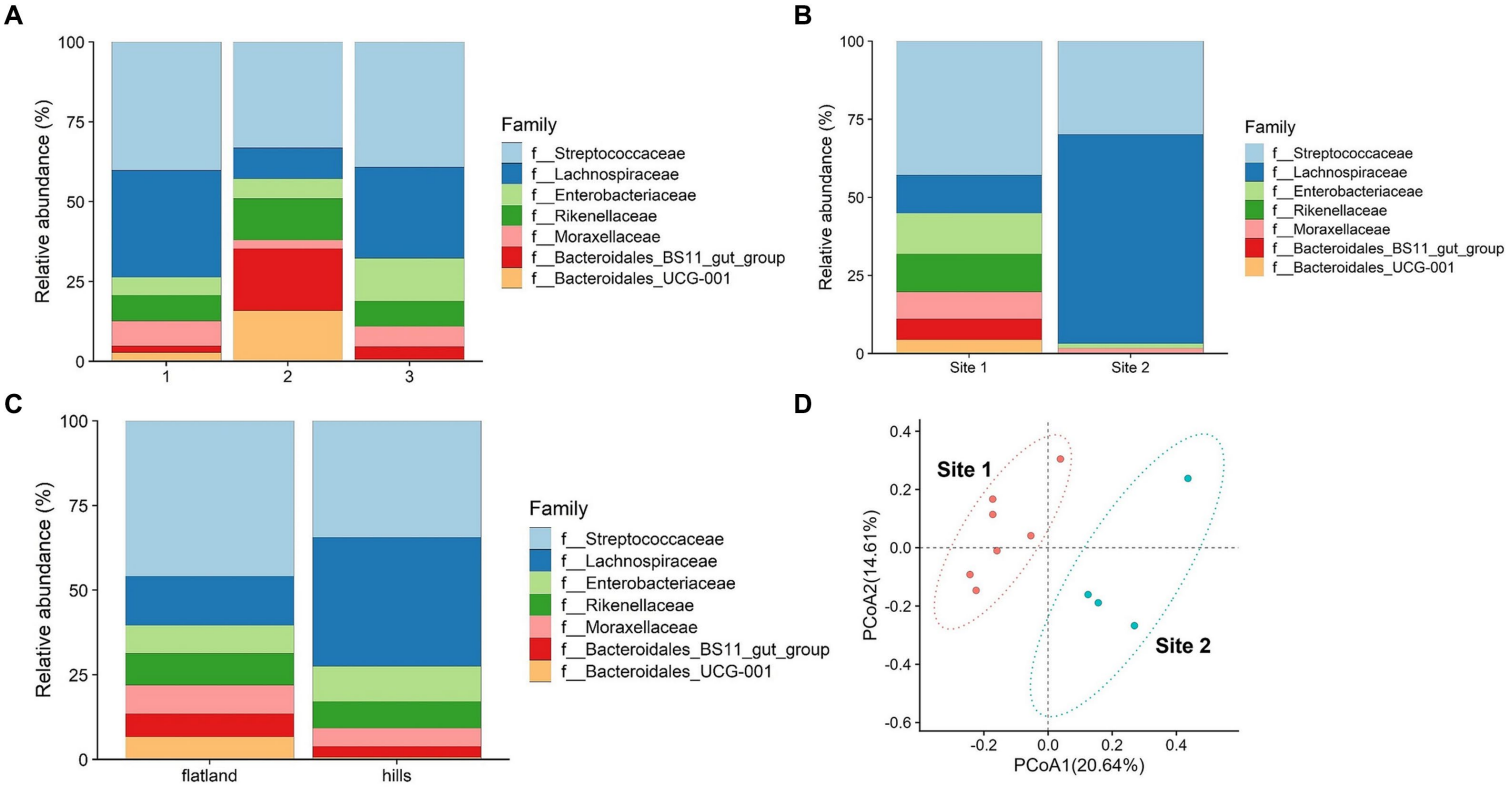
**(A)** Relative abundance of microbiota’s family divided into three groups of weight. Group 1 consisted of animals with an estimated weight below 400 kg, group 2 between 401 and 450 kg and group 3 above 451 kg. **(B)** Relative abundance of microbiota’s family divided into flatland and hills based on place of origin. **(C)** Relative abundance of microbiota’s family divided into the two conditioning sites. **(D)** Principal coordinate analysis of intestinal microbiota families comparing data from two conditioning sites.

The potential influence of estimated weight was considered. The animals were categorized into three groups based on their estimated weight percentile classes as indicated in Supplementary Table S1. Bacteroidales were significantly enriched in group 2 based on weight estimate (*p*-value = 0.02), whereas the Lachnospiraceae were more abundant in groups 1 and 3 (Figure 3A).

Bacteroidetes phyla are known for their vital roles in fiber digestion and organic acid synthesis. On the other hand, the Lachnospiraceae family contributes to the digestion and fermentation of complex plant polysaccharides (28), such as cellulose and hemicellulose, and are essential to produce volatile fatty acids (VFAs) that serve as an energy source (29, 30). Horses with the highest and lowest estimated weights exhibited a lower abundance of Bacteroidetes compared to the intermediate group, indicating that there might be a link between bacterial composition and energy utilization from the diet.

Previous studies have suggested that a decrease in Bacteroidetes abundance (31) and an increased ratio of Firmicutes to Bacteroides could potentially contribute to the promotion of fat deposition as an adaptive response (32). Furthermore, another research has indicated a positive association between high body mass index (BMI) and increased levels of Firmicutes (including Lachnospiraceae) and decreased levels of Bacteroidetes (33). In the context of the breed object of the study, the findings suggest that alterations in the relative abundance of these bacterial groups, with the changes in the proportions in group 3, may impact energy metabolism and contribute to the development of obesity. On the other hand, Firmicutes bacteria produce higher amounts of butyrate (34), which is recognized as a health-promoting molecule due to its ability to enhance insulin sensitivity (35). In specific circumstances, this increased production of butyrate by Firmicutes could potentially result in elevated energy expenditure and subsequent weight loss. Conversely, Lachnospiraceae were found to be less prevalent in group 2. Studies have shown higher levels of Lachnospiraceae in anorexic patients, and this bacterial presence remains unchanged even after short-term weight recovery (36). These findings underscore the importance of weight-related factors in influencing the composition of fecal microbiota, particularly the distinct roles played by Bacteroidetes and Lachnospiraceae-Firmicutes. Given the relatively limited number of animals involved, further investigation is necessary to gain a clearer understanding of the specific impact of the predominance of Firmicutes compared to Bacteroidetes on the body weight of horses.

The comparison between the place of origin where the horses were kept when they were foals, and their altitude (flatland and hills) showed statistically significant differences for the Lachnospiraceae family (*p*-value = 0.04), which could be attributed to the feeding strategies employed during the early years of the horses (Figure 3B). The type of breeding and feeding practices are known to leave a footprint on the animal’s microbiome (37, 38). Most of the flatland horses had limited access to pastures during their early years, while all the horses bred in the hills spent at least 6 months in a pasture. This difference in breeding practices may possibly explain the prevalence of Lachnospiraceae observed in the hills horse group. It is plausible to think that maybe the distinct composition of grass and hay in the pasture environment, which contains higher fiber content and is more resistant to degradation, requires a greater abundance of Lachnospiraceae to facilitate lignin degradation. Statistically significant differences were found comparing the two sampling locations (Site 1 and Site 2). Based on the Welch statistics, three families seem to be enriched in Site 1 and not in the other as shown in Supplementary Table S8. Enterobacteriaceae and Rikenellaceae were over-presented in Site 1, whereas Lachnospiraceae in Site 2 (Supplementary Table S8).

**Figure 4:**
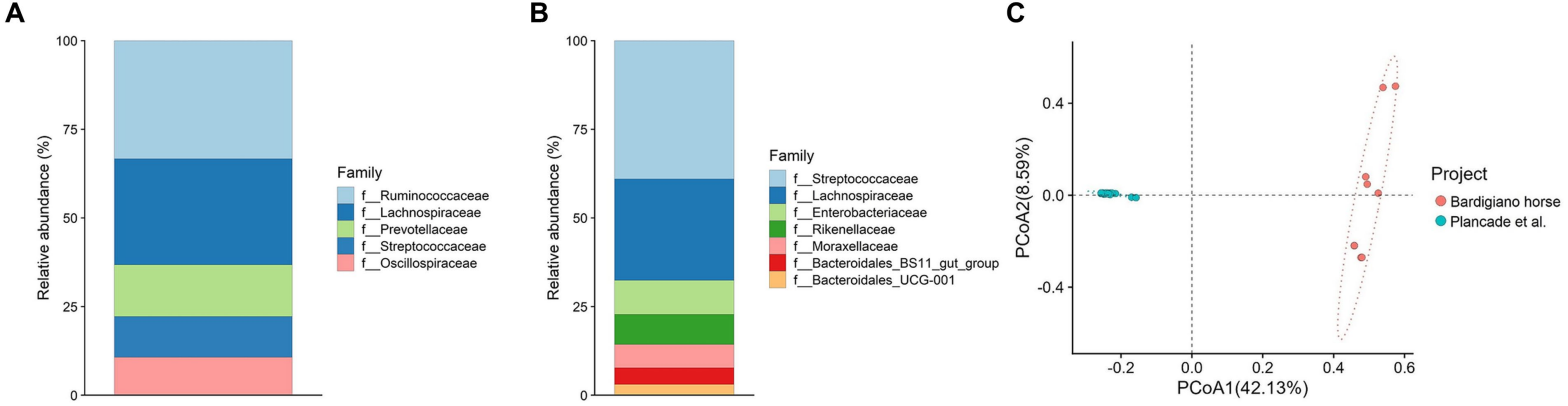
**(A,B)** Relative abundance of families comparing Bardigiano horses **(B)** with athletic breeds **(A)** from Plancade et al. (20). **(C)** Principal coordinate analysis of intestinal microbiota families comparing data from Bardigiano and athlete horses.

Dietary changes, stress, and environmental factors, such as training (25), have been shown to influence the composition of the microbiota in horses (39). As mentioned before, the two riding centers had different management practices. Figure 3C illustrates the abundance and types of bacterial families identified in the microbiota of the horses from the two riding centers. The observations belonging to each riding center cluster together and differ between the two centers, as shown in Figure 3D. At Site 1, a greater diversity of bacteria was observed, with a total of seven different families present. In contrast, Site 2 exhibited a reduced population with only four families, predominantly comprising Lachnospiraceae and Streptococcaceae. As previously mentioned, the horses at Site 2 were primarily fed hay with small amounts of concentrate feed, while at Site 1, the quantity of concentrate feed provided was three times higher. This disparity in diet composition likely contributed to the prevalence of Lachnospiraceae observed at Site 2, as reported by Zhu et al. (40). Thus, this could be an effect of feeding with a high percentage of hay, which contains fewer nutrients and degrades at a slower rate compared to a diet consisting of both hay and concentrate feed. This result shows that probably 70 days of conditioning protocol significantly impacted the horses’ fecal microbiota, due to the different feeding, training, and bedding type. Beta diversity performed with 999 permutations showed a significant difference (*p*-value = 0.05) among the two groups from different sites, confirming that environmental factors affect deeply microbiota composition (Figure 4A).

### 3.3 Comparison with athlete breeds

The comparison with athlete breeds showed that most abundant families differed between local and athletic horses, having in common Lachnospiraceae only as shown in Figures 4A,B.

Among the two populations, a statistically significant difference (*p*-value < 0.001) was found through the ANOVA test, which results are shown in Supplementary Table S9, suggesting that indeed breeds’ objectives play an important role in microbiota determination.

The Bray–Curtis dissimilarity index was calculated and used to perform a Pairwise Adonis test showing a significant difference (*p*-value < 0.001) in beta diversity among the two groups. Conversely from what has been suggested by Massacci et al. (18), the Bardigiano horse local breed clustered differently from the athletic breed mix in the PCoA as shown in Figure 4C.

### 3.4 Limits of the study

The limits of the study were the small number of mares due to the small size of the Bardigiano population. Experimenting with horses requires high expenses in handling and maintaining the subjects. No further metadata was available to deepen the comparison with the athlete breed. In addition, to fully comprehend the effect of the tested factors on the microbiota, the work would have benefit from a microbiota characterization at time 0 (before the conditioning period). However, due to limited budget this was not possible.

### 3.5 Further developments

Further developments of this study could concern the prevalence of antimicrobial resistance genes in Bardigiano horses compared to more common athlete breeds.

## 4 Conclusion

This is the first work aimed to describe the fecal microbiota of Bardigiano, an Italian local horse breed. Based on our findings, it seems that environmental changes in horse management impact the microbiota composition. At a broader taxonomic level, the analyzed samples resembled what has been seen in different horse breeds and attitudes in existing studies. However, at lower taxonomic levels, the distinction became clearer, suggesting distinguished characteristics of the Bardigiano breed. Comparison with the mix of athlete horse breeds reinforced these results. Despite the differences among the two breeding sites, Bardigiano horses clustered together when compare through PCoA against the athlete breeds: thus confirming these suggestions.

Understanding the microbiota composition in local breeds like the Bardigiano horse is important for preserving biodiversity, managing animal health, and promoting sustainable farming practices. It also helps to understand the complex relationship between animals and their microbial partners, leading to better strategies for animal welfare, conservation, and agricultural sustainability. Researches as this one are meaningful for the preservation of biodiversity, in alignment with the guidelines outlined by European directives and authorities involved in the safeguarding of biodiversity.

## Data availability statement

The datasets presented in this study can be found in online repositories. The names of the repository/repositories and accession number(s) can be found below: BioProject, PRJNA1001795.

## Ethics statement

The animal studies were approved by responsible for the welfare of animals at the University of Parma (protocol 14/CESA/2023). The studies were conducted in accordance with the local legislation and institutional requirements. Written informed consent was obtained from the owners for the participation of their animals in this study.

## Author contributions

AMCH, VA, and MA performed conceptualization, data curation, and writing – original draft preparation. AMCH performed data collection. ASa, ASu, and SB performed writing – review and editing. All authors contributed to the article and approved the submitted version.
