# Supplementary material for: Fecal microbiota characterization of an Italian local horse breed
Source: Front Vet Sci. 2024 Feb 15;11:1236476. doi: 10.3389/fvets.2024.1236476 (PMC10902133; doi:10.3389/fvets.2024.1236476)
Supplement: Supplementary file 1 [file Table_1.DOCX]

Supplementary Material

Microbiota characterization of an Italian local horse breed

Alicia Maria Carrillo Heredero, Alberto Sabbioni, Vittoria Asti^*^, Michela Ablondi, Andrea Summer, Simone Bertini

*** Correspondence:** Vittoria Asti [vittoria.asti@unipr.it](mailto:vittoria.asti@unipr.it)

**Table 1: Weight classes considered as variables**

| Weight class | Weight range (kg) |
| --- | --- |
| Group 1 | 350.00-400.99 |
| Group 2 | 401.00-450.99 |
| Group 3 | 451.00-550.00 |

Table 2: Relative abundance of phyla composing Bardigiano horse microbiota

| Phylum | Relative abundance (%) |
| --- | --- |
| Firmicutes | 50.21 |
| Bacteroidota | 33.52 |
| Spirochaetota | 6.27 |
| Fibrobacterota | 3.36 |
| Proteobacteria | 2.41 |
| Verrucomicrobiota | 1.64 |
| Actinobacteriota | 0.98 |
| Patescibacteria | 0.86 |
| Synergistota | 0.25 |
| Campylobacterota | 0.15 |
| un_k__Bacteria | 0.13 |
| Armatimonadota | 0.09 |
| Elusimicrobiota | 0.08 |
| Desulfobacterota | 0.05 |

Table 3: Relative abundance of classes composing Bardigiano horse microbiota

| Class | Relative abundance (%) |
| --- | --- |
| Clostridia | 39.30 |
| Bacteroidia | 33.52 |
| Bacilli | 6.52 |
| Spirochaetia | 6.27 |
| Negativicutes | 4.38 |
| Fibrobacteria | 3.36 |
| Gammaproteobacteria | 2.41 |
| Verrucomicrobiae | 1.64 |
| Saccharimonadia | 0.86 |
| Actinobacteria | 0.54 |
| Coriobacteriia | 0.44 |
| Synergistia | 0.25 |
| Campylobacteria | 0.15 |
| un_k__Bacteria | 0.13 |
| un_p__Armatimonadota | 0.09 |
| Elusimicrobia | 0.06 |
| Desulfovibrionia | 0.05 |
| Endomicrobia | 0.02 |

Table 4: Relative abundance of orders composing Bardigiano horse microbiota

| Order | Relative abundance (%) |
| --- | --- |
| Bacteroidales | 33.48 |
| Oscillospirales | 21.19 |
| Lachnospirales | 13.71 |
| Spirochaetales | 6.27 |
| Lactobacillales | 5.38 |
| Acidaminococcales | 3.83 |
| Fibrobacterales | 3.36 |
| Christensenellales | 2.73 |
| Verrucomicrobiales | 1.56 |
| Enterobacterales | 1.37 |
| Peptostreptococcales-Tissierellales | 1.09 |
| Pseudomonadales | 1.00 |
| Bacillales | 0.94 |
| Saccharimonadales | 0.86 |
| Veillonellales-Selenomonadales | 0.56 |
| Corynebacteriales | 0.49 |
| Coriobacteriales | 0.44 |
| Monoglobales | 0.30 |
| Synergistales | 0.25 |
| Erysipelotrichales | 0.20 |
| Clostridiales | 0.19 |
| Campylobacterales | 0.15 |
| un_k__Bacteria | 0.13 |
| Eubacteriales | 0.10 |
| un_p__Armatimonadota | 0.09 |
| Pedosphaerales | 0.08 |
| Elusimicrobiales | 0.06 |
| Micrococcales | 0.05 |
| Desulfovibrionales | 0.05 |
| Flavobacteriales | 0.04 |
| Xanthomonadales | 0.04 |
| Endomicrobiales | 0.02 |

Table 5: Relative abundance of families composing Bardigiano horse microbiota

| Family | Relative abundance (%) |
| --- | --- |
| Lachnospiraceae | 13.63 |
| Oscillospiraceae | 10.68 |
| Rikenellaceae | 8.39 |
| Prevotellaceae | 8.22 |
| F082 | 6.47 |
| Spirochaetaceae | 6.27 |
| Streptococcaceae | 4.63 |
| p-251-o5 | 4.33 |
| Acidaminococcaceae | 3.83 |
| UCG-010 | 3.49 |
| Fibrobacteraceae | 3.36 |
| [Eubacterium] coprostanoligenes group | 2.93 |
| Christensenellaceae | 2.73 |
| Ruminococcaceae | 2.68 |
| Bacteroidales UCG-001 | 2.35 |
| Bacteroidales BS11 gut group | 1.59 |
| Akkermansiaceae | 1.56 |
| Enterobacteriaceae | 1.35 |
| Hungateiclostridiaceae | 1.14 |
| Anaerovoracaceae | 1.09 |
| Moraxellaceae | 1.00 |
| Planococcaceae | 0.94 |
| Saccharimonadaceae | 0.86 |
| Lactobacillaceae | 0.74 |
| Paludibacteraceae | 0.69 |
| Bacteroidales RF16 group | 0.69 |
| Selenomonadaceae | 0.56 |
| Eggerthellaceae | 0.39 |
| Monoglobaceae | 0.30 |
| Synergistaceae | 0.25 |
| Nocardiaceae | 0.22 |
| M2PB4-65 termite group | 0.20 |
| Clostridiaceae | 0.19 |
| Bacteroidetes BD2-2 | 0.18 |
| gir-aah93h0 | 0.16 |
| Campylobacteraceae | 0.15 |
| Corynebacteriaceae | 0.14 |
| un_k__Bacteria | 0.13 |
| Marinifilaceae | 0.13 |
| Dietziaceae | 0.13 |
| Butyricicoccaceae | 0.12 |
| Erysipelatoclostridiaceae | 0.11 |
| Eubacteriaceae | 0.10 |
| Ethanoligenenaceae | 0.10 |
| un_p__Armatimonadota | 0.09 |
| Erysipelotrichaceae | 0.08 |
| Defluviitaleaceae | 0.08 |
| Pedosphaeraceae | 0.08 |
| Muribaculaceae | 0.07 |
| Elusimicrobiaceae | 0.06 |
| Desulfovibrionaceae | 0.05 |
| Coriobacteriales Incertae Sedis | 0.05 |
| [Clostridium] methylpentosum group | 0.04 |
| Weeksellaceae | 0.04 |
| Xanthomonadaceae | 0.04 |
| Promicromonosporaceae | 0.04 |
| Succinivibrionaceae | 0.02 |
| Micrococcaceae | 0.02 |
| Endomicrobiaceae | 0.02 |

Table 6: Relative abundance of genera composing Bardigiano horse microbiota

| Genus | Relative abundance (%) |
| --- | --- |
| Rikenellaceae RC9 gut group | 7.52 |
| un_f__F082 | 6.47 |
| Treponema | 6.25 |
| Streptococcus | 4.63 |
| Lachnospiraceae AC2044 group | 4.57 |
| NK4A214 group | 4.38 |
| un_f__p-251-o5 | 4.33 |
| Phascolarctobacterium | 3.83 |
| un_f__UCG-010 | 3.49 |
| un_f__Lachnospiraceae | 3.49 |
| Fibrobacter | 3.36 |
| un_f__[Eubacterium] coprostanoligenes group | 2.93 |
| Christensenellaceae R-7 group | 2.73 |
| UCG-002 | 2.72 |
| un_f__Bacteroidales UCG-001 | 2.35 |
| UCG-005 | 2.09 |
| Lachnospiraceae UCG-009 | 2.08 |
| Prevotellaceae UCG-001 | 1.87 |
| Prevotellaceae UCG-004 | 1.84 |
| Prevotella | 1.67 |
| un_f__Bacteroidales BS11 gut group | 1.59 |
| Akkermansia | 1.56 |
| Escherichia-Shigella | 1.35 |
| Alloprevotella | 1.21 |
| Ruminococcus | 1.15 |
| Prevotellaceae UCG-003 | 1.11 |
| Acinetobacter | 1.00 |
| Saccharofermentans | 0.96 |
| Candidatus Saccharimonas | 0.86 |
| Candidatus Soleaferrea | 0.82 |
| un_f__Paludibacteraceae | 0.69 |
| un_f__Bacteroidales RF16 group | 0.69 |
| hoa5-07d05 gut group | 0.68 |
| un_f__Ruminococcaceae | 0.67 |
| Lachnospiraceae XPB1014 group | 0.63 |
| Papillibacter | 0.60 |
| un_f__Oscillospiraceae | 0.55 |
| Solibacillus | 0.52 |
| un_f__Prevotellaceae | 0.49 |
| Ligilactobacillus | 0.48 |
| Oribacterium | 0.43 |
| un_f__Selenomonadaceae | 0.43 |
| Marvinbryantia | 0.37 |
| Lysinibacillus | 0.36 |
| Anaerovorax | 0.35 |
| Family XIII AD3011 group | 0.33 |
| Agathobacter | 0.33 |
| un_f__Eggerthellaceae | 0.32 |
| [Eubacterium] hallii group | 0.31 |
| Monoglobus | 0.30 |
| Blautia | 0.29 |
| Lachnospiraceae NK4A136 group | 0.28 |
| UCG-007 | 0.24 |
| Acetitomaculum | 0.23 |
| un_f__M2PB4-65 termite group | 0.20 |
| Mogibacterium | 0.19 |
| dgA-11 gut group | 0.19 |
| Clostridium sensu stricto 1 | 0.19 |
| un_f__Bacteroidetes BD2-2 | 0.18 |
| un_f__gir-aah93h0 | 0.16 |
| Campylobacter | 0.15 |
| Frisingicoccus | 0.14 |
| Ruminiclostridium | 0.14 |
| Corynebacterium | 0.14 |
| un_k__Bacteria | 0.13 |
| un_f__Marinifilaceae | 0.13 |
| Cloacibacillus | 0.13 |
| Dietzia | 0.13 |
| Family XIII UCG-001 | 0.12 |
| un_f__Synergistaceae | 0.12 |
| Gordonia | 0.12 |
| UCG-004 | 0.11 |
| Rhodococcus | 0.11 |
| Anaerosporobacter | 0.10 |
| Quinella | 0.10 |
| UCG-009 | 0.10 |
| un_f__Anaerovoracaceae | 0.10 |
| Eubacterium | 0.10 |
| Incertae Sedis | 0.10 |
| un_p__Armatimonadota | 0.09 |
| Lachnospiraceae UCG-010 | 0.09 |
| [Eubacterium] ruminantium group | 0.09 |
| Weissella | 0.09 |
| Defluviitaleaceae UCG-011 | 0.08 |
| DEV114 | 0.08 |
| possible genus Sk018 | 0.07 |
| un_f__Planococcaceae | 0.07 |
| Denitrobacterium | 0.07 |
| un_f__Muribaculaceae | 0.07 |
| Lactobacillus | 0.06 |
| Elusimicrobium | 0.06 |
| Pseudobutyrivibrio | 0.06 |
| HT002 | 0.06 |
| Lachnoclostridium | 0.06 |
| un_f__Lactobacillaceae | 0.05 |
| Phoenicibacter | 0.05 |
| Oscillibacter | 0.05 |
| Oscillospira | 0.05 |
| un_f__[Clostridium] methylpentosum group | 0.04 |
| Chryseobacterium | 0.04 |
| UCG-012 | 0.04 |
| Stenotrophomonas | 0.04 |
| Mailhella | 0.04 |
| Prevotellaceae Ga6A1 group | 0.04 |
| Catenisphaera | 0.04 |
| Isoptericola | 0.04 |
| un_f__Erysipelotrichaceae | 0.03 |
| Anaerovibrio | 0.03 |
| Pygmaiobacter | 0.02 |
| Caproiciproducens | 0.02 |
| UCG-008 | 0.02 |
| Lachnospiraceae ND3007 group | 0.02 |
| Succinivibrionaceae UCG-002 | 0.02 |
| [Anaerorhabdus] furcosa group | 0.02 |
| Arthrobacter | 0.02 |
| Endomicrobium | 0.02 |
| Sediminispirochaeta | 0.01 |
| Desulfovibrio | 0.01 |

Table 7: Relative abundance of species composing Bardigiano horse microbiota

| Species | Relative abundance (%) |
| --- | --- |
| un_g__Rikenellaceae RC9 gut group | 7.52 |
| un_f__F082 | 6.47 |
| un_g__Treponema | 6.20 |
| lutetiensis | 4.62 |
| un_g__Lachnospiraceae AC2044 group | 4.57 |
| un_g__NK4A214 group | 4.38 |
| un_f__p-251-o5 | 4.33 |
| un_g__Phascolarctobacterium | 3.71 |
| un_f__UCG-010 | 3.49 |
| un_f__Lachnospiraceae | 3.49 |
| un_g__Fibrobacter | 3.36 |
| un_f__[Eubacterium] coprostanoligenes group | 2.93 |
| un_g__Christensenellaceae R-7 group | 2.73 |
| un_g__UCG-002 | 2.72 |
| un_f__Bacteroidales UCG-001 | 2.35 |
| un_g__UCG-005 | 2.09 |
| un_g__Lachnospiraceae UCG-009 | 2.08 |
| un_g__Prevotellaceae UCG-001 | 1.87 |
| un_g__Prevotellaceae UCG-004 | 1.84 |
| un_g__Prevotella | 1.67 |
| un_f__Bacteroidales BS11 gut group | 1.59 |
| un_g__Akkermansia | 1.56 |
| coli | 1.31 |
| un_g__Alloprevotella | 1.21 |
| un_g__Prevotellaceae UCG-003 | 1.11 |
| lwoffii | 0.98 |
| un_g__Saccharofermentans | 0.96 |
| un_g__Candidatus Saccharimonas | 0.86 |
| un_g__Candidatus Soleaferrea | 0.82 |
| un_g__Ruminococcus | 0.71 |
| un_f__Paludibacteraceae | 0.69 |
| un_f__Bacteroidales RF16 group | 0.69 |
| un_g__hoa5-07d05 gut group | 0.68 |
| un_f__Ruminococcaceae | 0.67 |
| un_g__Lachnospiraceae XPB1014 group | 0.63 |
| un_g__Papillibacter | 0.60 |
| un_f__Oscillospiraceae | 0.55 |
| un_f__Prevotellaceae | 0.49 |
| un_g__Ligilactobacillus | 0.48 |
| un_g__Solibacillus | 0.45 |
| un_g__Oribacterium | 0.43 |
| un_f__Selenomonadaceae | 0.43 |
| flavefaciens | 0.39 |
| un_g__Marvinbryantia | 0.37 |
| un_g__Lysinibacillus | 0.36 |
| un_g__Anaerovorax | 0.35 |
| un_g__Family XIII AD3011 group | 0.33 |
| un_g__Agathobacter | 0.33 |
| un_f__Eggerthellaceae | 0.32 |
| un_g__[Eubacterium] hallii group | 0.31 |
| un_g__Monoglobus | 0.30 |
| un_g__Blautia | 0.29 |
| un_g__UCG-007 | 0.24 |
| un_g__Acetitomaculum | 0.23 |
| un_g__Lachnospiraceae NK4A136 group | 0.23 |
| un_f__M2PB4-65 termite group | 0.20 |
| un_g__Mogibacterium | 0.19 |
| un_g__dgA-11 gut group | 0.19 |
| un_f__Bacteroidetes BD2-2 | 0.18 |
| un_f__gir-aah93h0 | 0.16 |
| un_g__Frisingicoccus | 0.14 |
| un_g__Ruminiclostridium | 0.14 |
| un_k__Bacteria | 0.13 |
| un_f__Marinifilaceae | 0.13 |
| evryensis | 0.13 |
| un_g__Dietzia | 0.13 |
| un_g__Family XIII UCG-001 | 0.12 |
| succinatutens | 0.12 |
| un_f__Synergistaceae | 0.12 |
| un_g__Gordonia | 0.12 |
| un_g__UCG-004 | 0.11 |
| rhodochrous | 0.11 |
| un_g__Anaerosporobacter | 0.10 |
| un_g__Quinella | 0.10 |
| un_g__UCG-009 | 0.10 |
| butyricum | 0.10 |
| un_f__Anaerovoracaceae | 0.10 |
| un_g__Eubacterium | 0.10 |
| un_g__Incertae Sedis | 0.10 |
| un_p__Armatimonadota | 0.09 |
| un_g__Clostridium sensu stricto 1 | 0.09 |
| un_g__Lachnospiraceae UCG-010 | 0.09 |
| un_g__[Eubacterium] ruminantium group | 0.09 |
| cibaria | 0.09 |
| hyointestinalis | 0.08 |
| un_g__Defluviitaleaceae UCG-011 | 0.08 |
| glutamicum | 0.08 |
| un_g__DEV114 | 0.08 |
| un_g__possible genus Sk018 | 0.07 |
| un_f__Planococcaceae | 0.07 |
| un_g__Denitrobacterium | 0.07 |
| un_f__Muribaculaceae | 0.07 |
| un_g__Campylobacter | 0.07 |
| silvestris | 0.06 |
| equicursoris | 0.06 |
| un_g__Elusimicrobium | 0.06 |
| un_g__Pseudobutyrivibrio | 0.06 |
| un_g__HT002 | 0.06 |
| un_g__Lachnoclostridium | 0.06 |
| un_f__Lactobacillaceae | 0.05 |
| bacterium | 0.05 |
| un_g__Phoenicibacter | 0.05 |
| un_g__Oscillibacter | 0.05 |
| un_g__Oscillospira | 0.05 |
| saccharophilum | 0.05 |
| un_f__[Clostridium] methylpentosum group | 0.04 |
| stationis | 0.04 |
| un_g__Chryseobacterium | 0.04 |
| un_g__Escherichia-Shigella | 0.04 |
| un_g__UCG-012 | 0.04 |
| un_g__Stenotrophomonas | 0.04 |
| un_g__Mailhella | 0.04 |
| un_g__Prevotellaceae Ga6A1 group | 0.04 |
| un_g__Catenisphaera | 0.04 |
| un_g__Isoptericola | 0.04 |
| champanellensis | 0.03 |
| un_f__Erysipelotrichaceae | 0.03 |
| un_g__Anaerovibrio | 0.03 |
| un_g__Pygmaiobacter | 0.02 |
| un_g__Caproiciproducens | 0.02 |
| un_g__UCG-008 | 0.02 |
| un_g__Lachnospiraceae ND3007 group | 0.02 |
| un_g__Acinetobacter | 0.02 |
| un_g__Succinivibrionaceae UCG-002 | 0.02 |
| un_g__[Anaerorhabdus] furcosa group | 0.02 |
| gandavensis | 0.02 |
| un_g__Corynebacterium | 0.02 |
| un_g__Endomicrobium | 0.02 |
| bromii | 0.02 |
| un_g__Sediminispirochaeta | 0.01 |
| equinus | 0.01 |
| un_g__Desulfovibrio | 0.01 |

**Table 8: Enriched families from the comparison of the two conditioning sites.**

| Family | Enriched Group | Difference | P-value |
| --- | --- | --- | --- |
|  |  |  |  |
| Enterobacteriaceae | Site 1 | 0.115 | <0.001 |
| Lachnospiraceae | Site 2 | 0.546 | <0.001 |
| Rikenellaceae | Site 1 | 0.121 | <0.001 |

**Table 9: Results of ANOVA test on OTUs from the two breed groups.**

|  | Df | Sum Sq | Mean Sq | F value | P-value |
| --- | --- | --- | --- | --- | --- |
| Groups | 1 | 0.053421 | 0.053421 | 41.037 | <0.001 |
| Residuals | 29 | 0.037752 | 0.001302 |  |  |
